# Genomic Diversity Evaluation of *Populus trichocarpa* Germplasm for Rare Variant Genetic Association Studies

**DOI:** 10.3389/fgene.2019.01384

**Published:** 2020-01-28

**Authors:** Anthony Piot, Julien Prunier, Nathalie Isabel, Jaroslav Klápště, Yousry A. El-Kassaby, Juan Carlos Villarreal Aguilar, Ilga Porth

**Affiliations:** ^1^ Department of Wood and Forest Sciences, Université Laval, Quebec, QC, Canada; ^2^ Institute for System and Integrated Biology (IBIS), Université Laval, Quebec, QC, Canada; ^3^ Centre for Forest Research, Université Laval, Quebec, QC, Canada; ^4^ Natural Resources Canada, Canadian Forest Service, Laurentian Forestry Centre, Quebec, QC, Canada; ^5^ Scion, Rotorua, New Zealand; ^6^ Department of Forest and Conservation Sciences, Faculty of Forestry, University of British Columbia, Vancouver, BC, Canada; ^7^ Smithsonian Tropical Research Institute (STRI), Ancon, Panama; ^8^ Department of Biology, Université Laval, Quebec, QC, Canada

**Keywords:** annotation, genes, genetic architecture, missing heritability, rare defective alleles, small genetic variants, variant calling comparisons

## Abstract

Genome-wide association studies are powerful tools to elucidate the genome-to-phenome relationship. In order to explain most of the observed heritability of a phenotypic trait, a sufficient number of individuals and a large set of genetic variants must be examined. The development of high-throughput technologies and cost-efficient resequencing of complete genomes have enabled the genome-wide identification of genetic variation at large scale. As such, almost all existing genetic variation becomes available, and it is now possible to identify rare genetic variants in a population sample. Rare genetic variants that were usually filtered out in most genetic association studies are the most numerous genetic variations across genomes and hold great potential to explain a significant part of the missing heritability observed in association studies. Rare genetic variants must be identified with high confidence, as they can easily be confounded with sequencing errors. In this study, we used a pre-filtered data set of 1,014 pure *Populus trichocarpa* entire genomes to identify rare and common small genetic variants across individual genomes. We compared variant calls between *Platypus* and *HaplotypeCaller* pipelines, and we further applied strict quality filters for improved genetic variant identification. Finally, we only retained genetic variants that were identified by both variant callers increasing calling confidence. Based on these shared variants and after stringent quality filtering, we found high genomic diversity in *P. trichocarpa* germplasm, with 7.4 million small genetic variants. Importantly, 377k non-synonymous variants (5% of the total) were uncovered. We highlight the importance of genomic diversity and the potential of rare defective genetic variants in explaining a significant portion of *P. trichocarpa*'s phenotypic variability in association genetics. The ultimate goal is to associate both rare and common alleles with poplar's wood quality traits to support selective breeding for an improved bioenergy feedstock.

## Introduction

In tandem, phenotypic and genomic diversity assessments are key to understand the genetic regulation and architecture of quantitative traits. Genetic association studies in the form of genome-wide association studies (GWAS) have been used extensively to associate genome-wide polymorphisms to phenotypic variation (Visscher et al., 2017). Typical GWAS are only including common genetic variations. Most of these studies, however, failed to explain most of the observed heritability which is coined the missing heritability problem (Manolio et al., 2009; Brachi et al., 2011). It has been suggested that the missing heritability could be found in other forms of hereditary information such as epigenetic factors, epistasis, and rare genetic variation (Maher, 2008). For over a decade, human geneticists have questioned the role of rare genetic variants in complex diseases (Pritchard, 2001; McCarthy et al., 2008; Manolio et al., 2009). Consequently, the first association studies including rare genetic variants and the associated statistical tests originated in the field of human genetics (Cohen et al., 2004; Hoffmann et al., 2010; Wu et al., 2011).

Generally, most genetic polymorphisms in natural population are rare (*i.e.* found at frequencies lower than 5% in populations). In addition, deleterious variants tend to exist at low frequency in populations because of their negative impact on the phenotype. Non-synonymous genetic variants especially, may have important effects on phenotypes as they alter the amino acid sequence. For instance, a genetic variation leading to a stop codon gain can have drastic impacts on gene products (*i.e.* RNA and protein). Non-synonymous variants can either be missense or nonsense variants. Missense variants result in a codon change that code for a different amino acid while nonsense variants result in truncated or incomplete gene products. Including rare genetic variants in GWAS along with common genetic variants represents a unique opportunity to explain a significant part of the missing heritability (McClellan and King, 2010). Prior to genetic association studies, however, high confidence identification of the genetic polymorphisms within the studied population is required.

Due to their low frequency, rare genetic variants are challenging to identify. Genetic information for a substantial number of individuals is required to find those genetic variants that are rare in a population. In addition, rare genetic variants can easily be confounded with sequencing errors as high-throughput technologies have sequencing error rates between 0.1 to 1% (Fox et al., 2014). Therefore, rare genetic variants must be identified with high confidence before use in GWAS.

High-throughput sequencing permit the resequencing of large numbers of individuals at reasonable cost. Thanks to this technological advancement, genetic data for model species are now sufficiently large to identify rare genetic variants. Currently, the lack of computing resources remains one of the most important challenges to analyze these overwhelming data sets.

To decrease the confusion of low-frequency genetic variants with sequencing errors, strict quality filters are applied from processing of raw sequencing reads to variant discovery to discard bad quality reads and other chimeras. In addition, comparison between variant calling software resulting in a consensus set of Single Nucleotide Polymorphisms (SNP) lead to increased variant detection accuracy (Baes et al., 2014; Fahrenkrog et al., 2017). This approach minimizes the identification of false genetic variants, even though it will discard true genetic variants that were not identified by all variant callers. Using strict quality filtering and variant caller comparison, it is possible to evaluate both common and rare genetic diversity with high confidence. Sensitivity (the number of true positives) and specificity (the number of false positives) of the data processing and variant calling steps should be optimized according to the objectives of the genomic diversity evaluation.

Some populations are expected to contain a higher number of low-frequency genetic variants than others. Natural, outbreeding, and wide-ranging populations are expected to possess higher heterozygosity and a larger number of low-frequency variants (Petit and Hampe, 2006; Evans et al., 2014). On the contrary, domesticated species typically have reduced genetic diversity because of repeated cycles of artificial selection using a few performant breeders with common genetic backgrounds. Because of this high expected number of low-frequency genetic variants, natural forest tree species represent good candidates for rare variant association studies. In forest trees, rare nonsense variants associated to complex traits have been successfully identified. So far, these variants were found in the following genes and species: a *CAD* (*Cinnamyl alcohol dehydrogenase*) in *Pinus taeda* (MacKay et al., 1997), a *CCR* (*Cinnamoyl-CoA reductase*) in two *Eucalyptus* species (Thumma et al., 2005), an *HCT1* (*Hydroxycinnamoyl transferase*) in *Populus nigra* (Vanholme et al., 2013), and a *KANADI* in a *P. trichocarpa* x *P. deltoides* pseudo backcross (Muchero et al., 2015). Other studies also highlighted the ubiquity of rare genetic variants and their role in complex trait regulation in poplar species (Evans et al., 2014; Fahrenkrog et al., 2017).

**Figure 1:**
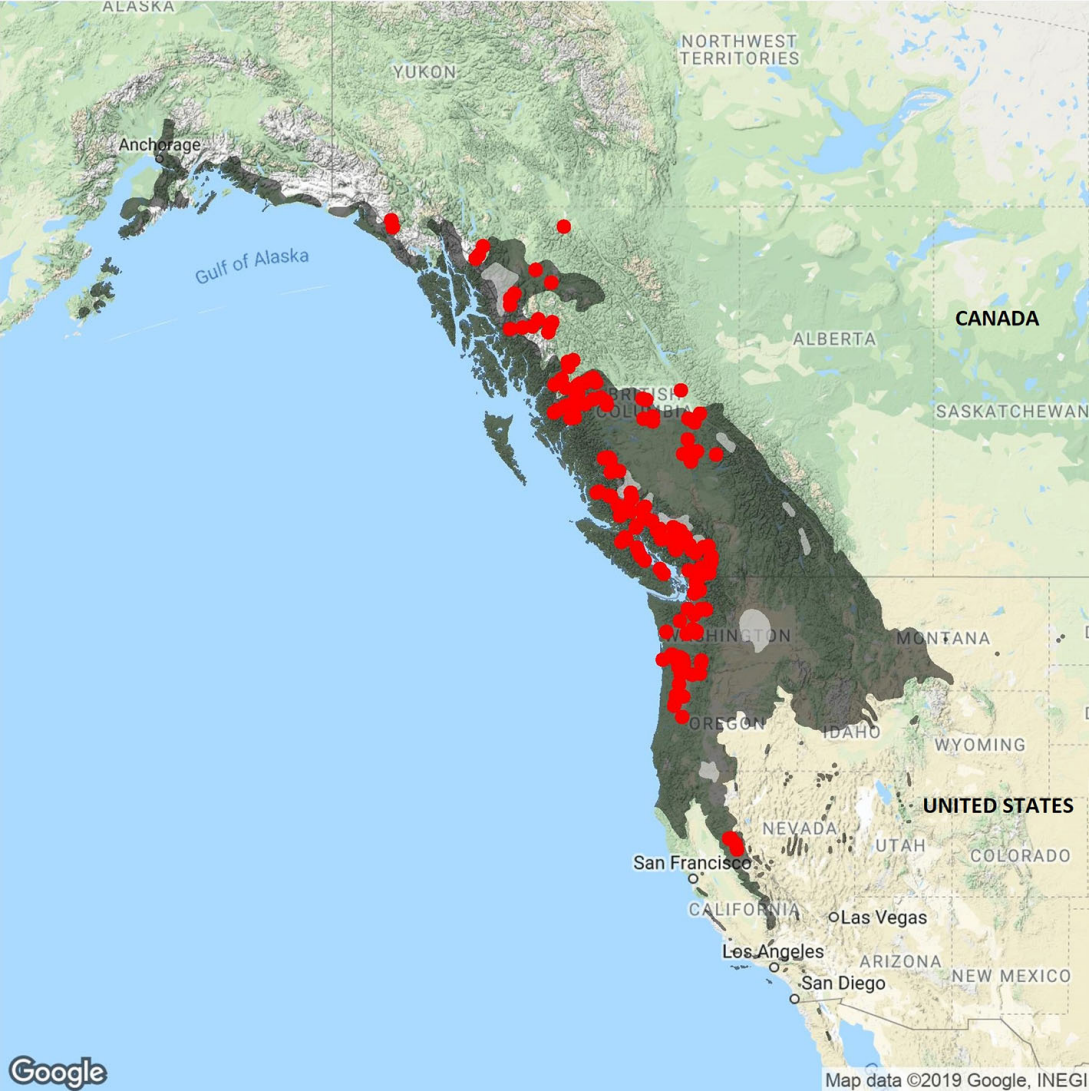
The 1,038 *P. trichocarpa* individuals retrieved from the JGI Genome Portal are represented by red dots across northwestern America*. P. trichocarpa* natural range is defined in dark grey. *P. trichocarpa* natural range was drawn from Little (1971).

*Populus trichocarpa* (Torr. & Gray), is a deciduous forest tree species with important ecological and economical aspects. This fast-growing tree mainly ranges along the North American west coast, from Alaska to Baja California Norte (latitude 31°N to 62°N) (**Figure 1**). The tree is used for pulp and oriented strand board production and represents a good candidate for second-generation biofuel feedstock (Porth and El-Kassaby, 2015). Additionally, *P. trichocarpa* was the first tree species to have its whole genome sequenced with a genome size close to 500Mbp (Tuskan et al., 2006). Since then, hundreds of whole genome resequencing efforts were conducted (Evans et al., 2014; Muchero et al., 2015; McKown et al., 2017) and numerous phenotypic traits related to phenology and wood properties have been measured in common garden experiments (Porth et al., 2013; Evans et al., 2014; McKown et al., 2014; Muchero et al., 2015).

Contrary to other better-studied model species, forest trees have not been subject to extensive genomic evaluation using whole genome resequencing data. Only a handful of such studies have been performed on economically important trees. Silva-Junior and collaborators used pooled resequencing of 240 Eucalyptus tree genome to develop a SNP chip able to identify 60K SNPs (Silva-Junior et al., 2015). *P. trichocarpa* is by far the forest tree species with the most available genetic resources. In 2014, Evans and colleagues evaluate the genomic diversity across a data set of 544 WGS of *P. trichocarpa* individuals identifying 17M variants (Evans et al., 2014). A second data set developed by the US Department Of Energy (DOE) BioEnergy Science Center (BESC) used 882 WGS of *P. trichocarpa* to identify 28M genetic variants genome-wide (https://bioenergycenter.org/besc/gwas/). To our knowledge, these few studies were the largest genomic evaluation studies performed to date using WGS. It must be noted that large number of individuals have been used in conifers to perform genomic evaluation, but these studies relied on exome or targeted sequencing constrained by the enormous and complex nuclear genome of these species. In 2016, Suren and collaborators used 579 interior spruce samples and 631 lodgepole pine samples to identify 10M SNPs and insertions/deletions (INDELs) in each species using exome capture (Suren et al., 2016).

The goal of the present study was to characterize the genomic diversity of *P. trichocarpa* individuals across its geographic range. The specific objective was to identifying low frequency genetic variants with high confidence that could be used in GWAS including both common and rare genetic variants. We present here a detailed evaluation of small genetic variants using strict quality filtering and comparison between two variant callers. In addition, we provide functional information obtained from the annotation of the discovered genetic variants. Finally, we performed a Gene Ontology (GO) enrichment of genes in which nonsense variants were found. This is the first study in a plant species aiming at rare allele discovery using a large sampling size from whole genome sequencing (over 1,000 individuals).

## Experimental Procedures

### *P. trichocarpa* Sequencing Reads

A total of 1,038 unique *P. trichocarpa* individuals were sequenced by the US DOE's BESC (Xie et al., 2009; Slavov et al., 2012). These individuals were sampled across most of *P. trichocarpa*'s geographic range in California, Oregon, and Washington, USA, as well as in British Columbia, Canada (**Figure 1**). These 1,038 *P. trichocarpa* accessions were retrieved online in fall 2017 from the Joint Genome Institute Genome Portal (https://genome.jgi.doe.gov/portal/) in the form of raw sequencing read files. Whole genome sequencing (WGS) were performed using short paired end reads (100 bp) on an Illumina HiSeq 2000 platform. The sampled individuals were checked for hybrids status after variant discovery based on comparison with closely related species in Principal Component Analysis (PCA; see Results).

### Sequencing Reads Quality Filtering

**Figure 2:**
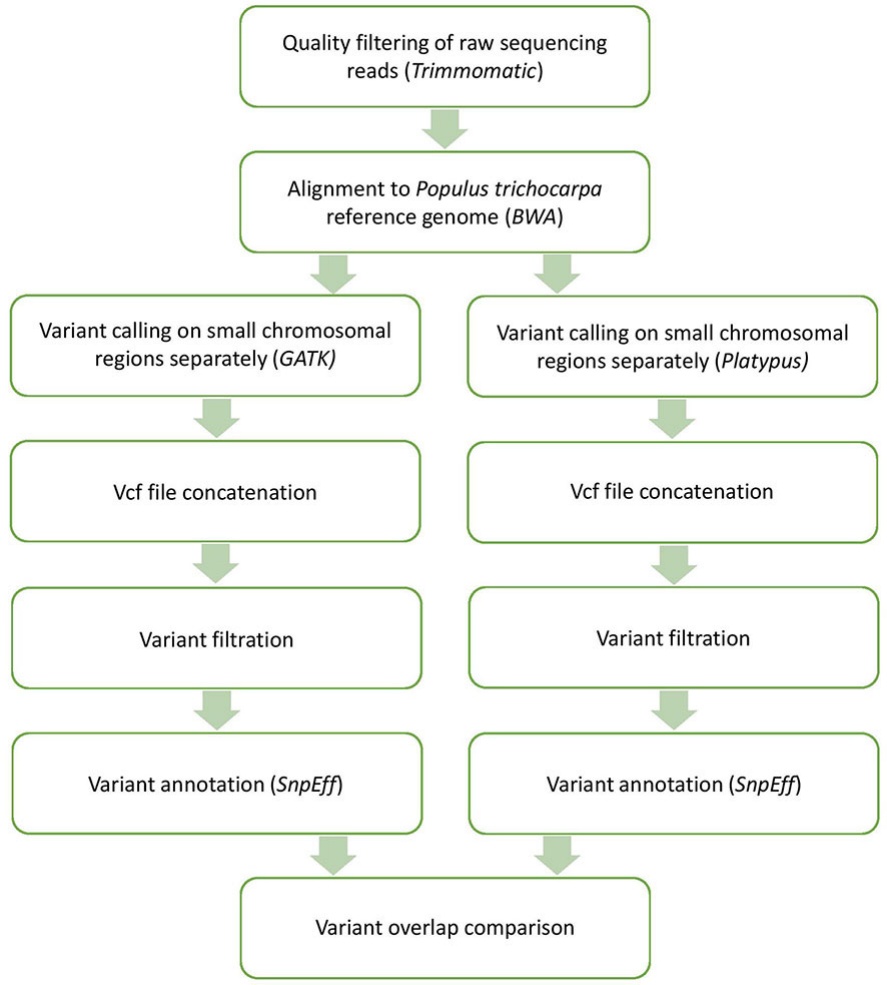
Methodology followed to obtain annotated filtered genetic variants from raw sequencing reads. Software used during each step are in parentheses.

Rare genetic variants and sequencing errors are both found at low frequencies in raw sequencing reads. To differentiate between true genetic variants and sequencing errors, we set stringent quality control on the raw read files. All bioinformatics manipulations were performed on *Cedar* and *Graham* computing servers from Compute Canada and on *Katak* and *Manitou* computing servers at the Institute of Integrative Biology and Systems, Université Laval (Quebec, Canada). First, we trimmed low-quality reads and sequencing adapters using *Trimmomatic* (Bolger et al., 2014) (**Figure 2**). Only bases having a Phred quality score higher than 27 (two chances out of 1,000 that the base is a sequencing error) were kept for further analyses. In addition, reads presenting a mean base quality score below 27 and/or shorter than 50 bases were discarded. The high quality of the cleaned read files was then ensured using *FastQC* (Andrew, 2010) before the alignment and variant calling steps. The mean number of paired reads per accession was about 66M (range: 24 to 321M) after quality filtering.

### Sequence Alignments

After the quality control steps, each individual accession was align to the reference genome of *P. trichocarpa* version 3.0 using the *Burrow Wheeler Aligner* (BWA; Li and Durbin, 2009) (**Figure 2**). We used the BWA-MEM algorithm that uses seedling alignments with maximal exact matches (MEMs) and then extending seeds with the affine-gap Smith-Waterman algorithm. Twenty-one genomes with average coverage lower than 5X were discarded in order to retain high confidence alignments. Ultimately, 1,017 alignments corresponding to the same number of unique individuals were used in the following analyses.

### Variant Calls From Two Different Software Pipelines

In order to obtain high confidence genetic variants, we used two types of variant calling software for result comparison (**Figure 2**): *Platypus* version 0.8.1 (Rimmer et al., 2014) and *HaplotypeCaller* from the *Genome Analysis Tool Kit* version 3.8 (*GATK*; DePristo et al., 2011; Poplin et al., 2017). These two variant calling software are widely used for variant discovery therefore facilitating data reproducibility. They also perform well in terms of sensitivity and precision of discovered variants while being computationally efficient thanks to the implementation of multithreading (Sandmann et al., 2017). *Platypus* enables the user to apply numerous quality filters during variant discovery, while *GATK* offers a filtering tool for use after variant discovery.

*Platypus* was used to perform single nucleotide variants (SNV) and INDEL calling on the 1,017 alignment files. As suggested by *Platypus* default parameter, bases with quality scores below 20 and reads with mapping quality below 20 were ignored during variant calling. The following custom parameters have been used to address rare variant calling: 1) only variants supported by at least 10 reads were considered; 2) reads having less than 40 bases with a quality lower than 20 were discarded; 3); variants where the median minimum quality in a window of 20 nucleotides around the variant fell below 20 were labelled as “bad reads”.

*HaplotypeCaller* was also used to perform SNV and INDEL calling on the 1,017 alignment files. The filtering tool *VariantFiltration* from *GATK* (DePristo et al., 2011) allowed us to apply quality filters to variants discovered by *HaplotypeCaller*. Parameters for filtering SNPs were set according to *GATK* recommendations for hard filtering. Variants were filtered out when: 1) their quality divided by nucleotide site depth was lower than 2; 2) they were located on a read with an approximate depth lower than 10; 3) their root mean square mapping quality was lower than 40; 4) their phred-scaled p-value using Fisher's exact test was greater than 60; their symmetric odds ratio of 2x2 contingency table to detect strand bias was greater than 3; their Z-score from Wilcoxon rank sum test of alternative vs. reference read mapping qualities was lower than -8; their z-score from Wilcoxon rank sum test of alternative vs. reference read position bias was lower than -12.5. In addition to the recommended parameters for hard-filtering, variants were filtered out if not supported by at least 10 reads.

Using custom python scripts, we filtered out *vcf* files obtained by *Platypus* and *HaplotypeCaller*. More precisely, variants that were attributed the “bad reads” flag were discarded from the *vcf* files obtained by the two types of software. Retained variants were therefore validated by each quality criteria settled during the variant calling phase. Additionally, only INDELs smaller than four nucleotides were included.

### Parallelization

**Figure 3:**
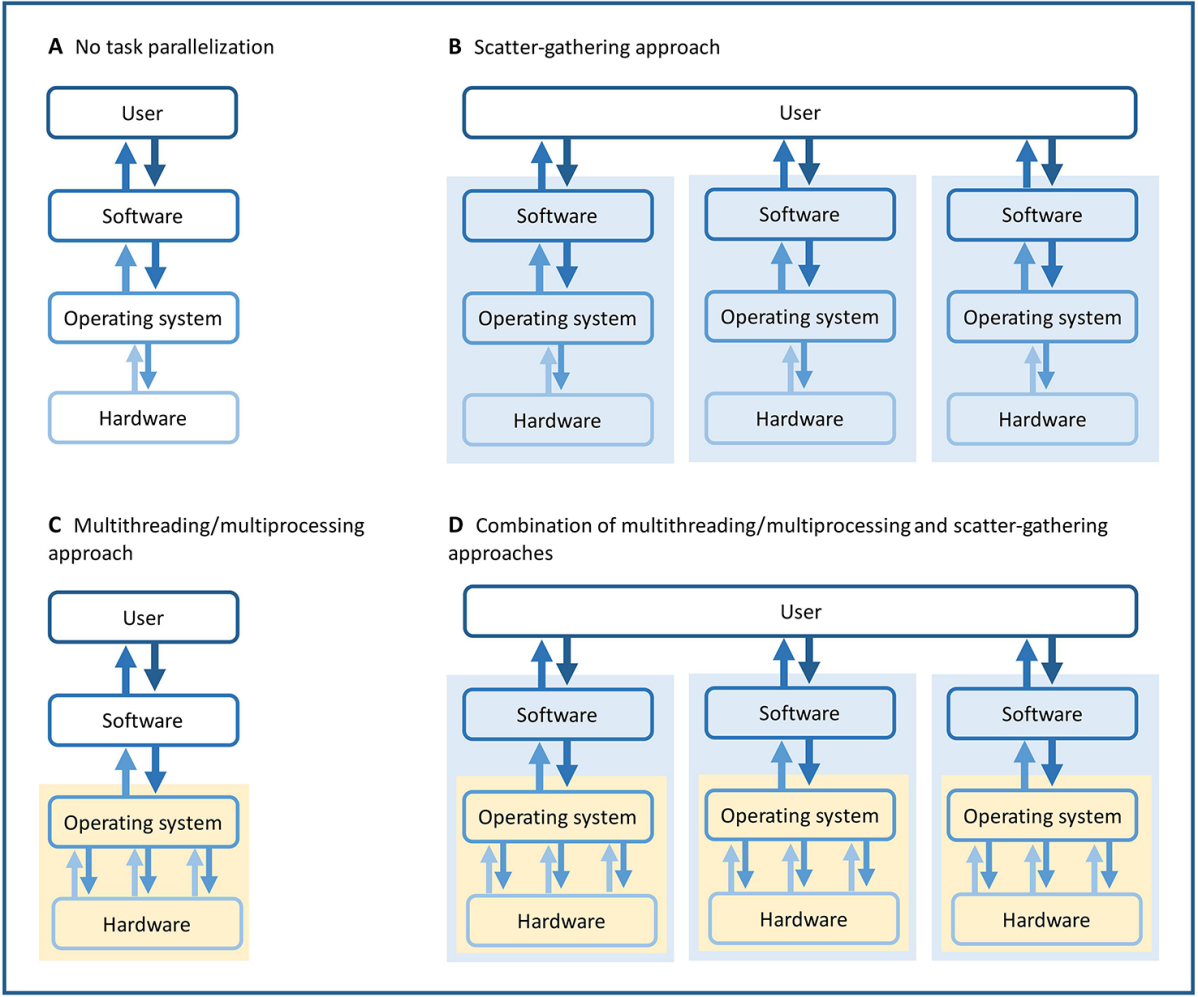
Illustration of different task parallelization approaches. **(A)** Simplest approach with no task parallelization. **(B)** Scatter-gathering approach, where task parallelization is done by the user and happens outside the software. **(C)** Multithreading/multiprocessing approaches, where task parallelization is done by and happens within the software. **(D)** Combination of multithreading/multiprocessing and scatter-gathering, where task parallelization happens outside and within the software. Yellow backgrounds highlight the multithreading and multiprocessing parallelization. Blue backgrounds highlight the scatter-gathering parallelization.

Given the large size of our data set, we took advantage of task parallelization in order to minimize computation time for the analyses of the two variant calling software (**Figure 3**). Both *Platypus* version 0.8.1 and *GATK's HaplotypeCaller* version 3.8 allow task parallelization within the software, using multiprocessing for *Platypus* and multithreading for *HaplotypeCaller*. In addition, we used task parallelization outside the software using a scatter-gathering approach. With this method, large files are divided into smaller regions (scattering) analyzed in parallel, then, the results are collected and merged together (gathering). Both approaches are based on task parallelization, but multiple tasks are run within the software using multiprocessing and multithreading, whereas task parallelization is done by the user and happens outside the software for the scatter-gathering approach. A combination of these two approaches was used in order to minimize both analysis and queue time on calculation servers. Portions of the genome were analyzed in parallel (scatter-gathering) while multiple tasks were also running in parallel on each portion (multiprocessing, multithreading). Using *Platypus*, we ran the analysis on each chromosome separately, while we had to divide the analyses on smaller chromosomal regions using *HaplotypeCaller*. The computing resources used for each analysis varied considerably according to the studied chromosomal regions.

### Variant Annotations

Filtered variants discovered by the two variant callers were annotated using *SnpEff* (Cingolani et al., 2012). To annotate variants based on the same reference genome used during read alignment we built a custom *SnpEff* database of the annotated genome of *P. trichocarpa* version 3.0.

### GO Enrichment

Based on variants recovered by the two variant calling software, we performed a gene ontology (GO) enrichment test using *PANTHER* version 14.1 (Mi et al., 2019). We retrieved the names of *P. trichocarpa* genes in which stop-gained genetic variants were found. Stop-gained variants can have drastic impact on phenotypes and have been found to affect wood composition in poplars (Muchero et al., 2015). We tested whether stop-gained variants are enriched in specific gene functions with respect to biological processes. *PANTHER* version 14.1 does not include *P. trichocarpa* annotations; therefore, we retrieved names of the closest *Arabidopsis thaliana* genes from *P*. trichocarpa genes possessing this type of nonsense variants. The closest *Arabidopsis* genes were determined during the annotation of the *P. trichocarpa* reference genome v3.1 by aligning *A. thaliana* TAIR10 proteins to the *P. trichocarpa* genome (the detailed procedure is available on the *P. trichocarpa* v3.1 Phytozome page). The closest *A. thaliana* gene can be found in the gene annotation file of the *P. trichocarpa* reference genome v3.1 (available on the JGI Genome Portal). This information is available for 84% of the *P. trichocarpa* genes. We used the *PANTHER* classification system to perform a statistical overrepresentation test in GO biological processes, using a Fisher's exact test with the names of *A. thaliana* genes most similar to the targeted *P. trichocarpa* genes. Fisher's exact test was used rather than the binomial test because the former assumes a hypergeometric distribution, which is more accurate for smaller gene lists. Finally, we applied False Discovery Rate (FDR) correction to the obtained p-values. FDR correction was designed to control the false positive rate in the statistical test results and is generally considered a better choice than Bonferroni correction in enrichment analysis (Mi et al., 2019).

## Results

### Variant Calling From Platypus and HaplotypeCaller

**Figure 4:**
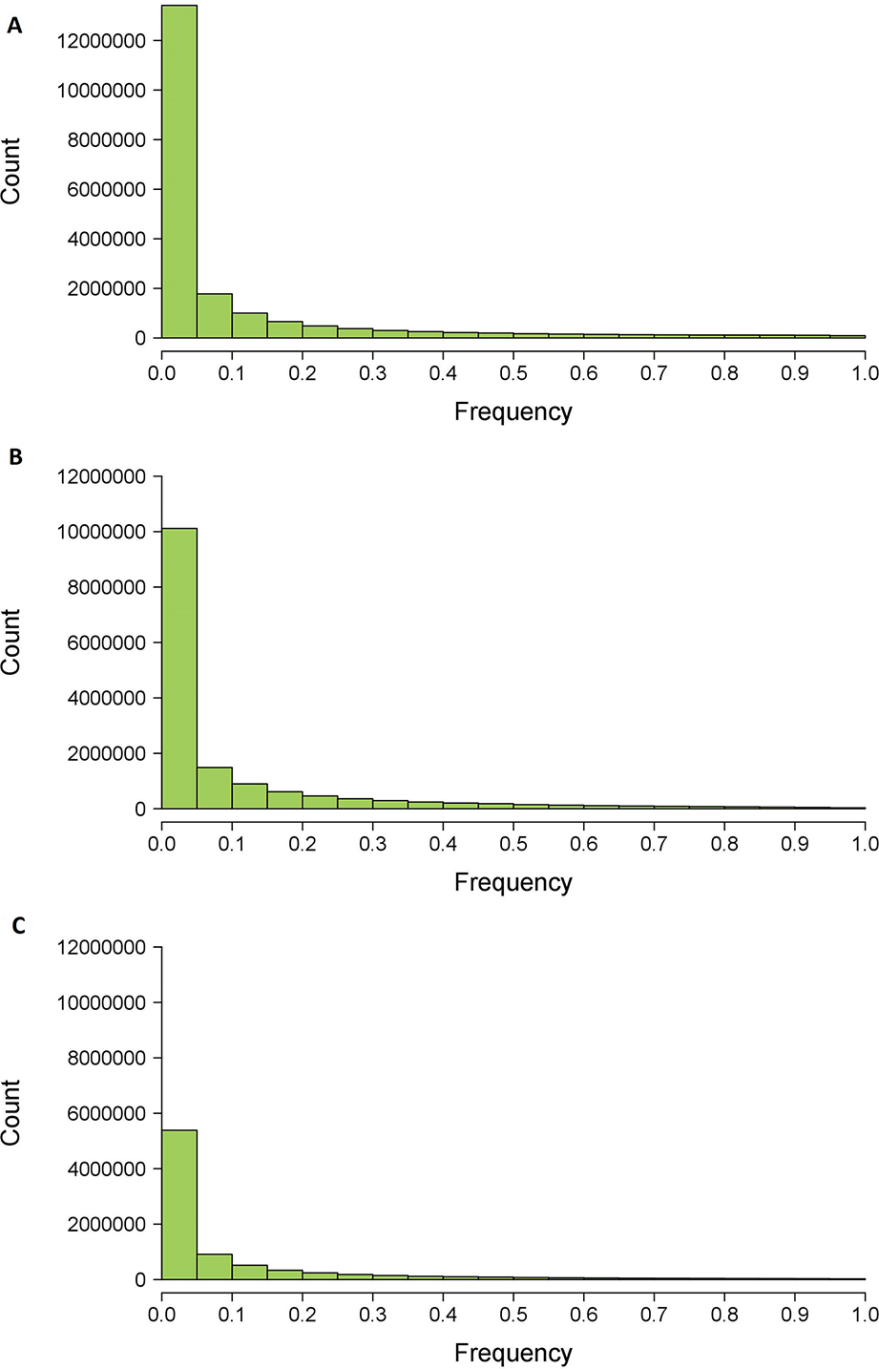
Histograms of variant frequencies after filtering from *HaplotypeCaller* **(A)**, *Platypus* **(B)** and consensus data set **(C)** between both software.

Before filtering, 31,607,230 genetic variants were identified by Platypus in our data set of 1,017 *P. trichocarpa* individuals. After filtering by quality and variant size, this number reduced to 15,734,785 variants, no longer than three consecutive nucleotides and distributed across 14,539,625 polymorphic sites. The majority of these variants (64%) showed a frequency in the population lower than 0.05 (**Figure 4**), *i.e.* found in less than 51 individuals.

Before filtering, 35,597,076 genetic variants have been identified by *HaplotypeCaller* across the 1,017 *P. trichocarpa* individuals. After filtering by quality and variant size, this number reduced to 19,971,499, no longer than three consecutive nucleotides and distributed across 19,478,954 polymorphic sites. Most of these variants (66%) had a frequency lower than 0.05 in the population (**Figure 4**).

### Variant Annotation

We used *SnpEff* to annotate genetic variants discovered by *Platypus* and *HaplotypeCaller.* Variant annotation uses information from reference genome annotations to describe genetic variants, such as the variants' inter- or intra-genic locations, and for variants located inside gene, the respective gene name and the effect of the variant on the entire nucleotide or gene coding sequence. We must note that the total number of variant annotation greatly exceed the total number of genetic variants. The reason is that some variants belong to more than one gene (*i.e.* overlapping genes) and here we report annotations for the effect of variants on each gene they belong to because the same variant can have different effects on different genes. On the contrary, we refer to the total number of genetic variants as the total number of nucleotide variation in the genome.

After annotation of variants discovered by *Platypus*, we found that most of the variants (86%) were located outside of genes, with nearly 11M variants found in intergenic regions and 8.1M and 7.8M variants found in upstream and downstream gene regions, respectively (**Table 1**). Upstream and downstream regions correspond to 5-kb-long regions around genes in *SnpEff* default parameter. The remaining variants (about 4.3M) were located in genic regions, with more than 633K non-synonymous variants (**Table 1**), accounting for 2% of the total.

**Table 1:** Annotations obtained from variant calling by *Platypus* and *HaplotypeCaller*.

Annotations	Current study			
	1,014 (1,017) individuals			
	Consensus		*Platypus*	*HaplotypeCaller*
Polymorphic sites	7,313,551	(8,368,838)	14,539,625	19,478,954
Total	7,441,340	(8,497,509)	15,734,785	19,971,499
intergenic variant^a^	5,254,503	(5,645,996)	10,886,077	15,149,344
downstream gene variant^b^	3,955,249	(4,607,452)	7,883,178	11,059,573
upstream gene variant^b^	3,955,094	(4,478,850)	8,086,954	11,413,237
intron variant^c^	1,341,551	(1,762,003)	2,427,258	3,463,071
missense variant^d^*	333,036	(418,974)	559,277	787,053
3 prime UTR variant^e^	269,591	(345,432)	484,519	672,092
synonymous variant^f^	231,894	(324,970)	410,776	554,853
5 prime UTR variant^e^	136,098	(175,634)	245,084	349,285
splice region variant^g^	54,271	(71,655)	95,479	128,316
5 prime UTR premature start gain^h^*	19,099	(24,989)	32,639	45,849
frameshift variant^i^*	9,766	(11,103)	16,937	31,172
stop gained^j^*	8,365	(9,226)	12,967	20,146
splice donor variant^k^*	2,694	(3,208)	4,387	6,237
splice acceptor variant^k^*	2,284	(2,689)	3,807	5,315
stop lost^l^*	1,082	(1,335)	1,994	2,612
start lost^m^*	821	(981)	1,511	2,123
stop retained variant^n^	535	(695)	925	1,246
initiator codon variant°	115	(142)	215	280
non_coding_transcript_variant^p^	66	(70)	368	221
intragenic_variant^q^	2	(5)	13	21
exon loss variant^r^	2	(3)	3	3
5 prime UTR truncation^s^	2	(2)	2	2
non canonical start codon^t^	1	(1)	2	2
3 prime UTR truncation^s^	0	(1)	1	1

For the consensus data set, numbers in brackets indicate the number of variants before suspected hybrids removal, while the number outside the brackets indicates the number of variants after suspected hybrids were already removed.*Non-synonymous variants corresponding to genetic variants inside coding regions altering the amino acid sequence of a protein and identified in both caller analyses. ^a^Intergenic variant: located in intergenic regions and outside upstream and downstream gene regions. ^b^Upstream and downstream variant: located in 5kb regions before and after a gene, respectively. ^c^Intron variant: located in non-translated introns of genes. ^d^Missense variant: located inside coding regions and resulting in an amino acid change. ^e^5 and 3 prime UTR variant: located in 5′ and 3′ untranslated region of a gene, respectively. ^f^Synonymous variant: located inside coding regions and not resulting in an amino acid change. ^g^Splice region variant: located within the region of the splice site. ^h^5 prime UTR premature start gain: resulting in an initiator codon inside the 5′ untranslated region. ^i^Frameshift variant: resulting in a reading frame change, because the number of nucleotides inserted or deleted is not a multiple of three. ^j^Stop gained: resulting in a premature stop codon in the coding sequence. ^k^Splice donor and acceptor variant: changing the 2 nucleotide regions at the 5′ and 3′ end of an intron, respectively. ^l^Stop lost: resulting in an elongated gene product because of stop codon loss. ^m^Start lost: resulting in initiator codon loss. ^n^Stop retained variant: change in one base in the terminator codon, but the terminator remains. °Initiator codon variant: change in at least one base of the first codon of a transcript. ^p^Non-coding transcript variant: located in a non-coding RNA gene. ^q^Intragenic variant: occurs within a gene but falls outside of all transcript features. ^r^Exon loss variant: resulting in the loss of an exon from a transcript. ^s^5 and 3 prime UTR truncation: causing the reduction of the 5′ and 3′ untranslated region, respectively. ^t^Non-canonical start codon: a start codon that is not the usual AUG sequence. The total number of variant annotations does not equal the total number of variants. The reason is that some variants are part of several overlapping genes and may have different effect on different genes.

For annotation of variants discovered by *HaplotypeCaller*, we found that most of the variants were located outside of genes, with 15M variants found in intergenic regions and 11.4M and 11M variants found in upstream and downstream gene regions, respectively (**Table 1**), accounting for 86.1% of the total. The remaining variants (about 6.1M) were located in genic regions, with nearly 901K non-synonymous variants (**Table 1**), accounting for 2.1% of the total.

### Variant Calling Overlap

**Figure 5:**
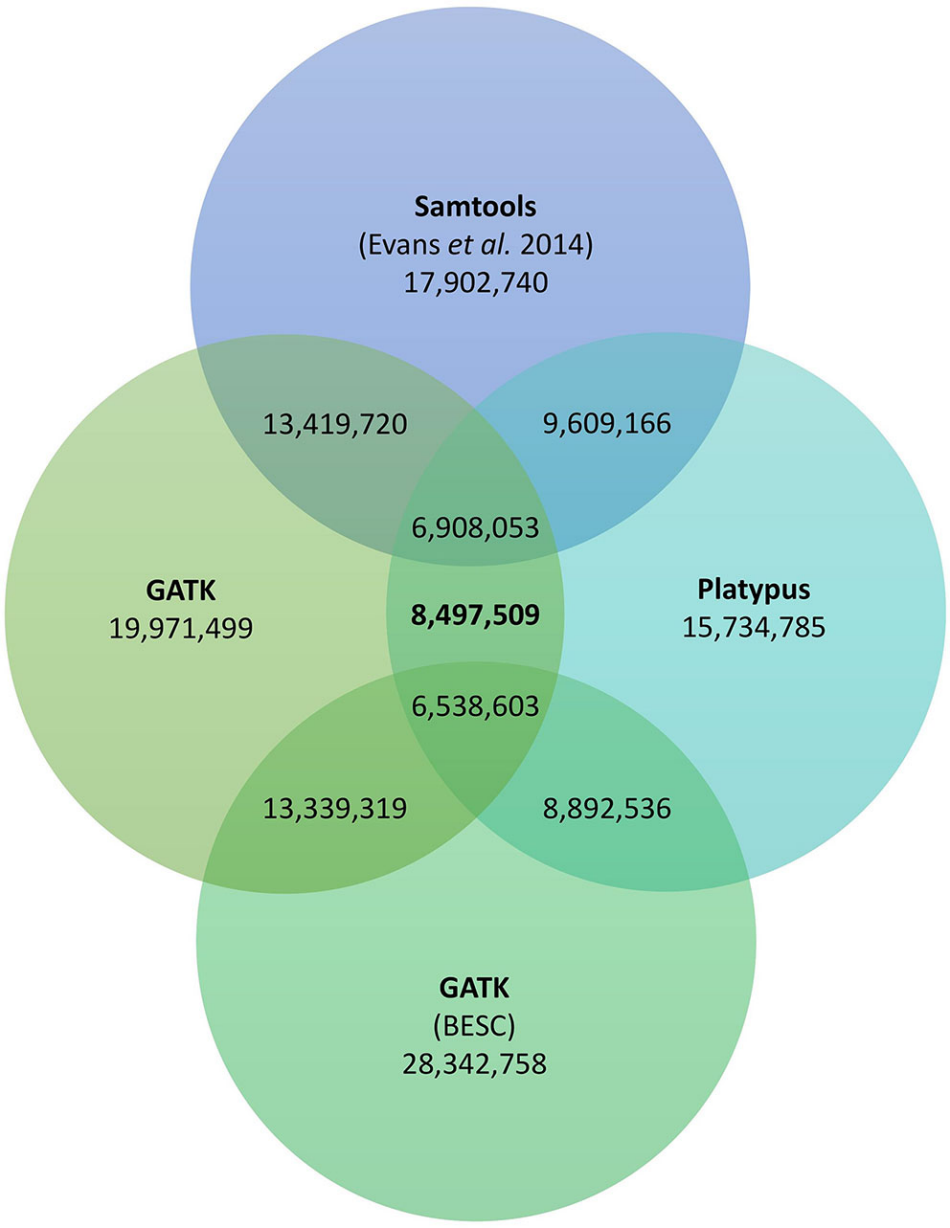
Venn diagram of the number of genetic variants identified in this study in addition to variants identified by Evans and colleagues (Evans et al., 2014) and variants in the BESC data set (https://bioenergycenter.org/besc/gwas/). We used *Platypus* and *GATK's HaplotypeCaller* with 1,017 WGS of *P. trichocarpa* individuals. Evans et al., 2014 used *samtools* and *bcftools* conjointly and 544 WGS of *P. trichocarpa* individuals. The BESC data set was called using multiple tools from *GATK* and 882 WGS of *P. trichocarpa* individuals. The number of variants identified by more than one software are indicated where circles overlap. We note here that some of the individuals used in Evans et al. and the BESC data set are the same as the 1,017 used in the present study.

In order to add a further quality criterion to the filtering process of genetic variants, we retained only variants recovered by the two variant calling software used in this study (*i.e. Platypus* and *Haplotype Caller*). We used the *isec* command from *bcftools* to find common variants between the two *vcf* files leading to a consensus variant set (Li, 2011). As a result, 8.5M genetic variants were recovered by both variant calling software, distributed across 8.4M polymorphic sites (**Figure 5**).

Most of the variants were located outside of genes, with 5.7M variants found in intergenic regions and 4.6M and 4.5M variants found in downstream and upstream gene regions, respectively (**Table 1**), accounting for 82.4% of the total. The remaining variants (3.2M) were located in genic regions, with nearly 473K non-synonymous variants (**Table 1**), accounting for 2.6% of the total. Missense variants (419K) accounted for 2.3% of the total and nonsense variants (54K) for 0.3% of the total.

We found 45% more non-synonymous variants compared to synonymous variants. Furthermore, among these non-synonymous variants, missense variants even exceeded synonymous variants by 29%. The total number of genetic variants was only 1.5% higher than the number of polymorphic sites.

To explore result disparities between genomic evaluation studies that used different methods and data set sizes, we also identified the genetic variants commonly found between our study and two other genomic evaluation studies on *P. trichocarpa* (Evans et al., 2014; https://bioenergycenter.org/besc/gwas/). When comparing our results with the study of Evans and collaborators that used 544 P*. trichocarpa* individuals and *Samtools* as a variant caller we found that 81% (6,908,053) of the variants they identified are also present in our consensus data set. When comparing our results with the BESC data set that used 882 P*. trichocarpa* individuals and tools from *GATK* we found that 77% (6,538,603) of the variants they identified are also present in our consensus data set (**Figure 5**). Interestingly, we found less variants in common with the study using 882 individuals comparing to the study using only 544 individuals. Details regarding individual SNP sets from the two variant callers overlap with the SNPs from Evans et al. and the BESC data set and a summary indicating which variants occur within each SNP set are provided in the Supplement (**Tables S1** and **S2**).

### Hybrid Identification

**Figure 6:**
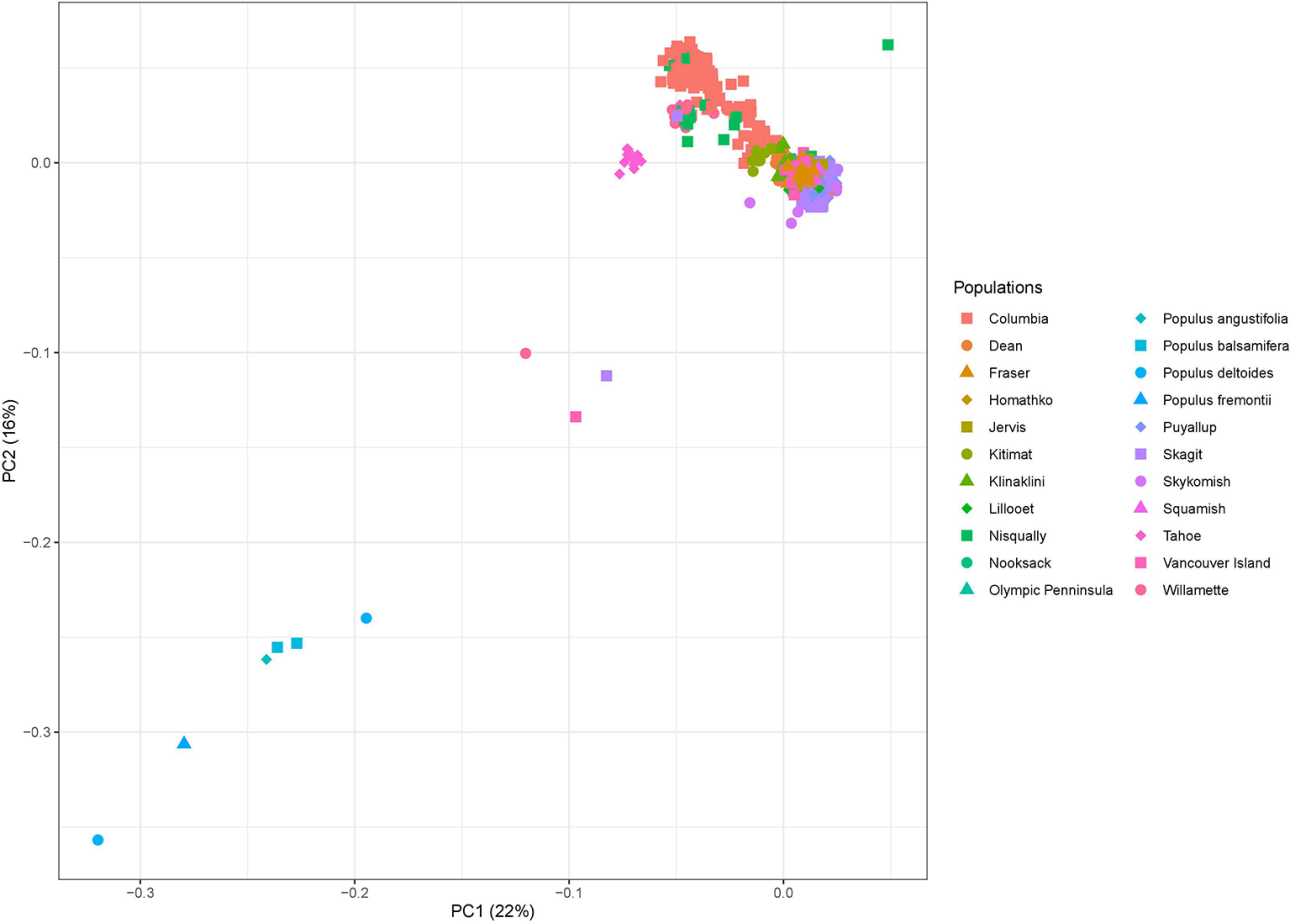
Principal component analysis showing the first two principal components of the genetic variation found across 1,017 *P. trichocarpa*, two *P. balsamifera*, two *P. deltoides*, one *P. angustifolia,* and one *P. fremontii* individual genomes retrieved from various databases for comparison. Note Nisqually-1 (Tuskan et al., 2006) in the upper right corner used as the overall reference.

In order to identify potential hybrids in our data set, we also identified the genetic variation across two *Populus balsamifera*, two *Populus deltoides*, one *Populus angustifolia,* and one *Populus fremontii* individuals for comparison. These species are closely related to *P. trichocarpa* and co-occur naturally in some parts of its natural range (Wang et al., 2019). These four species therefore hybridize naturally with *P. trichocarpa*. Raw WGS reads were downloaded from the JGI Genome Portal for *P. balsamifera* and *P. deltoides* (https://genome.jgi.doe.gov/portal/) and from the Genome Sequence Archive of the BIG Data Center for *P. angustifolia* and *P.* fremontii (https://bigd.big.ac.cn/gsa/, accession number CRA001510). Genetic variants from this six genomes were identified with the same bioinformatic pipeline used for *P. trichocarpa* individuals (see Experimental procedures). We used visual identification from a PCA to identify potential *P. trichcocarpa* hybrids. To do so, we filtrated the genetic variants identified across the genomes of these four species and the consensus variant set identified in this study using *plink* (–geno 0.01 –maf 0.1 –hwe 0.01 –LD 50 10 0.1) (Purcell et al., 2007). This filtration step yielded 12,001 variants with which we performed the PCA using *plink* (–pca 2) (**Figure 6**).

The fractions of the genetic variation explained by the two PCAs were 22% and 16%, respectively. Graphical representation of Principal Components (PC) 1 and 2 (**Figure 6**) clearly separated *P. balsamifera, P. deltoides*, *P. angustifolia* and *P. fremontii* from the 1,017 individuals of our consensus variant set. Every individual from the Tahoe population was slightly separated from the core of *P. trichocarpa* individuals. The Tahoe population is the southernmost population of our data set, geographically quite distant from the other *P. trichcocarpa* populations. This suggests that individuals from the Tahoe population differ genetically from other *P. trichocarpa* populations because of geographic distance and not because of hybridization with other *Populus* species. One individual each, from the Skagit, Vancouver Island and Willamette populations, respectively, were located halfway between the core of *P. trichocarpa* individuals and other *Populus* species, strongly suggesting that these individuals may be hybrids or introgressed. These three individuals were therefore removed from the consensus variant set, hence lowering the number of *P. trichocarpa* individuals to 1,014.

### Biological Pathways Overrepresented Among Functionally Defective Alleles

Among the consensus variants from *Platypus* and *GATK* variant calling (based on the finalized 1,014 individuals data set), we found that 8,365 stop-gained variants were distributed in 6,327 *P. trichocarpa* genes. These genes corresponded to 3,829 synonymous genes for *A. thaliana*. Analyses of gene function classification in *PANTHER* show that the set of genes containing stop-gained variants was enriched in 106 GO terms with respect to biological processes. Interestingly, multiple GO terms related to wood properties such as cell wall polysaccharide metabolism, cellulose biosynthesis, phenylpropanoid metabolism and plant-type cell wall biogenesis are enriched in genes possessing stop-gained variants (**Table 2**).

**Table 2:** Results from the Gene ontology (GO) enrichment test performed with PANTHER are presented using a list of *A. thaliana* genes closest to the *P. trichocarpa* genes and related to wood formation and in which stop-gained variants where found querying 1,014 black cottonwood individuals.

GO biological complete	List of genes with stop-gained variants					
	#	Expected	Enrichment	+/−	raw P-value	FDR
cellulose biosynthetic process	20	6.66	3	+	1.19E-04	8.89E-03
beta-glucan biosynthetic process	22	8.33	2.64	+	2.69E-04	1.82E-02
glucan biosynthetic process	36	15.69	2.29	+	4.52E-05	4.28E-03
cellular polysaccharide biosynthetic process	49	23.6	2.08	+	2.56E-05	2.55E-03
macromolecule metabolic process	1092	792.57	1.38	+	1.15E-26	1.14E-23
organic substance metabolic process	1556	1145.6	1.36	+	1.82E-39	5.41E-36
metabolic process	1798	1334.13	1.35	+	5.28E-47	3.15E-43
organic substance biosynthetic process	452	370.95	1.22	+	6.11E-05	5.13E-03
biosynthetic process	475	393.99	1.21	+	9.41E-05	7.69E-03
cellular biosynthetic process	441	363.73	1.21	+	1.09E-04	8.44E-03
cellular metabolic process	1487	1109.93	1.34	+	3.52E-34	4.20E-31
cellular process	2021	1610.26	1.26	+	8.40E-36	1.67E-32
cellular macromolecule metabolic process	823	606.54	1.36	+	1.10E-17	7.31E-15
cellular polysaccharide metabolic process	73	39.57	1.85	+	9.51E-06	1.07E-03
polysaccharide metabolic process	93	60.67	1.53	+	2.86E-04	1.91E-02
carbohydrate metabolic process	188	138.27	1.36	+	1.38E-04	1.02E-02
primary metabolic process	1430	1049.26	1.36	+	9.69E-36	1.45E-32
cellular carbohydrate metabolic process	92	56.64	1.62	+	5.83E-05	5.04E-03
polysaccharide biosynthetic process	51	28.6	1.78	+	5.06E-04	2.99E-02
cellular carbohydrate biosynthetic process	53	28.74	1.84	+	1.77E-04	1.27E-02
cellular glucan metabolic process	56	29.57	1.89	+	5.06E-05	4.51E-03
glucan metabolic process	56	29.57	1.89	+	5.06E-05	4.44E-03
cell wall polysaccharide metabolic process	39	19.16	2.04	+	1.99E-04	1.38E-02
plant-type cell wall biogenesis	41	20.41	2.01	+	1.89E-04	1.33E-02
cell wall biogenesis	56	31.93	1.75	+	3.56E-04	2.24E-02
cellular component biogenesis	228	171.45	1.33	+	8.33E-05	6.90E-03
cellular component organization or biogenesis	547	426.48	1.28	+	2.54E-08	4.59E-06
phenylpropanoid metabolic process	34	17.08	1.99	+	8.21E-04	4.62E-02
organic cyclic compound metabolic process	548	369.84	1.48	+	1.69E-17	1.01E-14
cellular aromatic compound metabolic process	518	355.81	1.46	+	2.52E-15	1.37E-12

Results are sorted hierarchically to better understand the hierarchical relations between over-represented functional classes. We provide for each GO term (up to seven levels): the number of genes present within the analyzed list (#), the expected number of genes under no GO enrichment (Expected), the enrichment value (Enrichment), the sign of the enrichment (+/−), the P-value associated with the enrichment test without multiple testing correction (raw P-value) and multiple testing corrected using False Discovery Rate (FDR).

## Discussion

Tool comparison for genomic variant calling has become the standard when using Next Generation Sequencing in clinical diagnostics (Sandmann et al., 2017, *e.g.*). To our knowledge, this approach has never been used in plant sciences when performing a large scale genomic diversity evaluation using WGS. In our study, we evaluated the genomic diversity across 1,017 individuals of *P. trichocarpa* in the form of small genetic variation using an existing set of whole genome sequences. Our goal was to identify rare and common genetic variation in the form of SNPs and small INDELs for subsequent use in GWAS. Using stringent filtering steps and variant calling comparison between two software we identified a set of high confidence genetic variants.

### Performance Comparison Between Platypus and HaplotypeCaller

Our data set was computationally heavy with more than one thousand *P. trichocarpa* genomes (~450 Mbp). For this reason, we opted to use variant calling software enabling multithreading to speed up variant identification analyses. *HaplotypeCaller* from *GATK* version 3.8 was considerably slower at identifying variants compared to *Platypus* version 0.8.1. Multithreading for current versions of *GATK* (version 4) is still under development and not safe for production work, therefore, we used a previous version of *GATK* (version 3.8). Both software identify variants based on haplotype reconstruction while *Platypus* also integrates a Bayesian statistical framework for variant discovery. The two software ran on the same data set, but the number of variants identified between each software differed substantially. *HaplotypeCaller* identified 27% more variants in comparison to *Platypus*. This discrepancy in the number of variants identified by the two variant calling software highlight the importance of result comparison between variant callers.

### Bioinformatic Approaches

We used a scatter-gathering approach coupled to multithreading to perform variant calling on smaller parts of the data separately. We either conducted the variant calling on chromosomes or smaller chromosomal regions separately to reach acceptable running time and computing resource use. This approach allowed us to identify variants across more than one thousand complete genomes of *P. trichocarpa* within reasonable time. An approach based on a single thread would not have permitted to reach our goal with current computing technologies. The combination of multithreading and scatter-gathering proved very efficient for variant discovery on a large data set.

### Consensus Variant Set

Nearly 8.5M genetic variants were identified by the two software and represent high confidence genetic variation. The vast majority of the identified variants had a frequency lower than 0.05 in our data set. Our results are in close agreement with other genetic diversity evaluation studies of *P. trichocarpa* or closely related species (Evans et al., 2014; Fahrenkrog et al., 2017) and is expected in outcrossing, wide ranging, and undomesticated tree populations (Petit and Hampe, 2006; Fahrenkrog et al., 2017). Most genetic variants are located outside the gene space where nucleotide substitutions are expected to have lower effect on the phenotype and therefore are less subject to purifying selection.

### Non-Synonymous/Synonymous Variant Ratio

More surprisingly, inside coding regions, non-synonymous genetic variants were more numerous than synonymous mutations. This pattern has already been observed in a similar study on *P. trichocarpa* (Evans et al., 2014). Given their higher impact on protein sequence, purifying selection is expected to be stronger on non-synonymous variants compared to synonymous ones. A positive ratio of non-synonymous to synonymous genetic substitutions is associated with positive selection (Yang and Bielawski, 2000). *P. trichocarpa* is wide-ranging across the west coast of North America and across a large latitudinal gradient from Alaska to southern California. Individuals in our data set were collected across most of *P. trichocarpa*'s range. Consequently, individuals in this study adapted to different environmental conditions and likely exhibit high genetic diversity in response to local adaptation (Evans et al., 2014). Populations genomic studies are needed to evaluate selection pressures and especially adaption acting across *P. trichocarpa* geographic range.

### Comparison With Other Genomic Evaluations on Poplars

Previous studies evaluated the genomic diversity in *P. trichocarpa* (Evans et al., 2014; BESC SNP data set: https://bioenergycenter.org/besc/gwas/) and *Populus deltoides (* Fahrenkrog et al., 2017). Fahrenkrog and colleagues (2017) used targeted resequencing and variant calling overlap between three different software to identified 358K SNPs in 391 unrelated individuals of *P. deltoides*, which is much lower than the 8.5M variants we found. Their final data set included variants found in a subset of genes, thus reducing the size of the analyzed genome. Intergenic variants were also excluded while most genetic variations are usually found in intergenic regions. Moreover, the variant calling comparison between three different software further decreased the number of identified variants. This approach resulted in a set of high confidence rare and common genetic variants, although less numerous than for studies based on WGS.Using 544 WGS of *P. trichocarpa* individuals, Evans and colleagues (2014) identified 17M SNPs using one variant caller. This number is more than two times higher than the 8.5M variants we identified using comparison between two variant calling software and stringent quality criteria on two times the number of individuals. Evans and colleagues performed no variant filtration, however, and found that stringent filtering had minimal impact on the sensitivity of known SNP discovery while reducing substantially the number of known SNPs passing the filtering threshold (*i.e.* specificity). For the targeted identification of rare genetic variants and for sequencing data with low to moderate sequencing depth we believe that variant filtration is highly beneficial. The DOE's BESC also released a SNP data set (a description of how the SNPs were called is available in the method section of the following study: Weighill et al., 2018). This data set included 28M variants identified across 882 WGS of *P. trichocarpa.* Genetic variants were called using *GATK* tools. First, variants were called independently for each individual using *HaplotypeCaller* and merged afterward. Biallellic SNPs were then extracted and filtered using the *VariantQualityScoreRecalibration* (VQSR) tool. This latter tool uses machine learning to filter variants using a set of known genetic variants (see Weighill et al., 2018 for more information). Similarly, to Evans and colleagues, the BESC data set identified a lot more genetic variants than our study using less individuals. The number of identified variants seems to increase when using only one variant caller. On the contrary, using variant caller comparison the number of individuals scanned does not seem to increase the number of identified variants. Indeed, the number of common variants between our study and the 882 individual data set is slightly lower than the number of common variants between our study and the 544 individuals data set (**Figure 5**). The number of commonly identified variants can even be greater between two different variant callers than between the same variant caller, *i.e. HaplotypeCaller.* These observations show that the use of a certain variant caller is not the main factor determining which variants will be identified, instead parameters used during variant discovery and for filtering along with the comparison between variant caller seem to be of considerable importance.

### Quality Filtering and Variant Caller Comparison

Application of stringent filtering criteria before and after variant discovery and the result overlap between variant calling software are key factors for genomic diversity evaluation. With current sequencing technologies and variant calling algorithms, a balance must be found between sensitivity and specificity of variant discovery. Increasing severity in quality filters and increasing the number of variant calling software tend to increase the quality of the identified variants while decreasing the total number of variants. Therefore, the goal of genomic diversity evaluation studies must be clearly stated to ensure that optimal parameters for variant identification are used. Common genetic variants can be identified easily with high confidence without using strict quality filters or comparison between variant calling software. On the contrary, rare genetic variants are difficult to identify with high confidence and require strict quality filtering and overlap between results from various variant calling softwares for reliable identification. When identifying both common and rare genetic variants, as in this study, confidence in the identified variants should be prioritized.

### Predicting Models for Increased Specificity

When high quality sets of genetic variants are already available as in model species, one can build models to better detect true and false genetic variants using sets of known genetic variants to increase the specificity of variant identification [*e.g. VQSR* from *GATK* (McKenna et al., 2010)]. Although these models are very useful for human and some other model species, they do not apply to every study. Large sequence data sets such as WGS and Whole Exome Sequencing (WES) and high quality sets of known genetic variants must be used in order to build accurate predicting models. WGS and WES are now widely used in *P. trichocarpa* and high quality sets of known common genetic variants are available. Known high quality sets of rare genetic variants, however, are scarce or even non-existent when considering both genic and intergenic regions. Consequently, we did not use such models to increase the specificity of our variant discovery. The consensus set of 8.5M genetic variants, common and rare, identified in this study will be available as a high quality set of known variants to build models aiming at increasing variant specificity in future genomic diversity evaluations of *P. trichocarpa* and closely related species.

### GO Enrichment

We used a GO enrichment test to identify biological pathways overrepresented with genes containing stop-gained genetic variants. A multitude of biological process were overrepresented with genes containing stop-gained variants. Among them, biological processes related to wood properties, and especially secondary cell-wall polysaccharides are of great interest. Previous studies already highlighted the role of functional variants (premature or abolished stop codon, altered start codon, frameshift variant or alternative splice sites) on genes involved in lignin biosynthesis (MacKay et al., 1997; Thumma et al., 2005; Vanholme et al., 2013; Muchero et al., 2015). The lignin and other secondary cell-wall polymers (*i.e.* cellulose and hemicellulose) biosynthesis pathways may be largely affected by functional variants. Thus, these overrepresented biopathways will help us select candidate genes for further analyses. Recent functional mutations are expected to show greater effects on a phenotype, since such functional allelic variants have not undergone selection to much extent. For example, we detected a stop gain mutation at 2.3% minor allele frequency and with 4.3% carriers in the population for the poplar orthologue of the Arabidopsis *irx10* gene (aka *PtrGUT2B*; Potri.001G068100). Its protein is known to be implicated in xylan backbone formation, and thus a prime target for improving cell wall traits (Porth et al., 2018). Therefore, such variants are important candidates for the purpose of rare variant association studies and ultimately, selective breeding with rare defective alleles (Vanholme et al., 2013; Porth and El-Kassaby, 2015).

## Conclusion

We identified 8.5M small genetic variants, common and rare, across more than one thousand *P. trichocarpa* individuals sampled throughout the species' range. Use of appropriate quality filtering and variant comparison between two variant callers resulted in high-quality sets of genetic variants. With a data set of 1,017 complete genomes, this is the first time that a genomic diversity evaluation of this magnitude has been conducted in *P. trichocarpa* and, to our knowledge, in any tree species. The high-quality set of known genetic variants identified will be directly available to support other genomic diversity evaluations of *P. trichocarpa* and other closely related species. Moreover, GWAS including rare and common genetic variants will be conducted using those high-quality variants. Thus, starting out from a wealth of genetic variants uncovered in the present study, we will be able to further narrow down the set of important variants for poplar selective breeding.

## Data Availability Statement

Raw sequence data are available under https://phytozome.jgi.doe.gov/pz/portal.html.

## Author Contributions

AP obtained data, analyzed all data, and wrote the manuscript. JP supported data analysis. NI, JK, and YE-K provided valuable insights on poplar genomics. JA obtained co-funding. IP designed the study, obtained funding, and helped in drafting the manuscript. All authors read and approved the manuscript.

## Conflict of Interest

Author JK was employed by the company New Zealand Forest Research Institute Limited (Scion).

The remaining authors declare that the research was conducted in the absence of any commercial or financial relationships that could be construed as a potential conflict of interest.

The reviewer LZ and handling Editor declared their shared affiliation.
