# Supplementary material for: Genomic Diversity Evaluation of Populus trichocarpa Germplasm for Rare Variant Genetic Association Studies
Source: Front Genet. 2020 Jan 28;10:1384. doi: 10.3389/fgene.2019.01384 (PMC6997551; doi:10.3389/fgene.2019.01384)
Supplement: Supplementary file 1 [file Table_1.docx]

**Table S1**. Annotations obtained from variant callings by *Platypus* and *HaplotypeCaller*, our consensus dataset, and the comparisons with two other genomic evaluations on *P. trichocarpa*.

**Legend**

For the consensus dataset, numbers in brackets indicate the number of variants before suspected hybrids removal, while the number outside the brackets indicates the number of variants after suspected hybrids were already removed.*Non-synonymous variants corresponding to genetic variants inside coding regions altering the amino acid sequence of a protein and identified in *both* caller analyses. ^a^We provide a comparison with a variant calling study also performed on *P. trichocarpa* by Evans and colleagues (Evans et al. 2014). ^b^We also provide a comparison with a variant calling study performed by the BioEnergy Science Center (BESC) on *P. trichocarpa*. ^c^Intergenic variant: located in intergenic regions and outside upstream and downstream gene regions. ^d^Upstream and downstream variant: located in 5kb regions before and after a gene, respectively. ^e^Intron variant: located in non-translated introns of genes. ^f^Missense variant: located inside coding regions and resulting in an amino acid change.

^g^5 and 3 prime UTR variant: located in 5’ and 3’ untranslated region of a gene, respectively. ^h^Synonymous variant: located inside coding regions and not resulting in an amino acid change. ^i^Splice region variant: located within the region of the splice site. ^j^5 prime UTR premature start gain: resulting in an initiator codon inside the 5’ untranslated region. ^k^Frameshift variant: resulting in a reading frame change, because the number of nucleotides inserted or deleted is not a multiple of three. ^l^Stop gained: resulting in a premature stop codon in the coding sequence.

^m^Splice donor and acceptor variant: changing the 2 nucleotide regions at the 5' and 3’ end of an intron, respectively. ^n^Stop lost: resulting in an elongated gene product because of stop codon loss. ^o^Start lost: resulting in initiator codon loss. ^p^Stop retained variant: change in one base in the terminator codon, but the terminator remains. ^q^Initiator codon variant: change in at least one base of the first codon of a transcript. ^r^Non-coding transcript variant: located in a non-coding RNA gene. ^s^Intragenic variant: occurs within a gene but falls outside of all transcript features. ^t^Exon loss variant: resulting in the loss of an exon from a transcript. ^u^5 and 3 prime UTR truncation: causing the reduction of the 5' and 3’ untranslated region, respectively. ^v^Non-canonical start codon: a start codon that is not the usual AUG sequence. The total number of variant annotations does not equal the total number of variants. The reason is that some variants are part of several overlapping genes and may have a different effect on different genes.

| **Annotations** | **Current study** | | | | **Evans et al. 2014^a^** | **BESC^b^** |
| --- | --- | --- | --- | --- | --- | --- |
|  | **1,017 individuals** | | | | **544 individuals** | **882 individuals** |
|  | **Overlap** | | ***Platypus*** | ***HaplotypeCaller*** | ***Samtools*** | ***GATK*** |
| Polymorphic sites | 7,313,551 | (8,368,838) | 14,539,625 | 19,478,954 | N.D. | 28,342,758 |
| Total | 7,441,340 | (8,497,509) | 15,734,785 | 19,971,499 | 17,902,740 | 28,342,758 |
| intergenic variant^c^ | 5,254,503 | (5,645,996) | 10,886,077 | 15,149,344 | 14,520,224 | 19,282,463 |
| downstream gene variant^d^ | 3,955,249 | (4,607,452) | 7,883,178 | 11,059,573 | N.D. | 13,074,290 |
| upstream gene variant^d^ | 3,955,094 | (4,478,850) | 8,086,954 | 11,413,237 | N.D. | 13,055,571 |
| intron variant^e^ | 1,341,551 | (1,762,003) | 2,427,258 | 3,463,071 | 1,962,848 | 3,388,134 |
| missense variant*^f^ | 333,036 | (418,974) | 559,277 | 787,053 | 612,655 | 1,573,776 |
| 3 prime UTR variant ^g^ | 269,591 | (345,432) | 484,519 | 672,092 | N.D. | 473,800 |
| synonymous variant ^h^ | 231,894 | (324,970) | 410,776 | 554,853 | 386,103 | 1,227,374 |
| 5 prime UTR variant ^g^ | 136,098 | (175,634) | 245,084 | 349,285 | N.D. | 237,037 |
| splice region variant ^i^ | 54,271 | (71,655) | 95,479 | 128,316 | N.D. | 192,871 |
| 5 prime UTR premature start gain* ^j^ | 19,099 | (24,989) | 32,639 | 45,849 | N.D. | 32,060 |
| frameshift variant* ^k^ | 9,766 | (11,103) | 16,937 | 31,172 | N.D. | 0 |
| stop gained* ^l^ | 8,365 | (9,226) | 12,967 | 20,146 | 18,702 | 118,792 |
| splice donor variant* ^m^ | 2,694 | (3,208) | 4,387 | 6,237 | 4,449 | 20,687 |
| splice acceptor variant* ^m^ | 2,284 | (2,689) | 3,807 | 5,315 | 3,748 | 20,601 |
| stop lost* ^n^ | 1,082 | (1,335) | 1,994 | 2,612 | 2,175 | 4,654 |
| start lost* ^o^ | 821 | (981) | 1,511 | 2,123 | 1,631 | 4,090 |
| stop retained variant ^p^ | 535 | (695) | 925 | 1,246 | 959 | 3,087 |
| initiator codon variant ^q^ | 115 | (142) | 215 | 280 | N.D. | 3,347 |
| non_coding_transcript_variant ^r^ | 66 | (70) | 368 | 221 | N.D. | 0 |
| intragenic_variant ^s^ | 2 | (5) | 13 | 21 | N.D. | 0 |
| exon loss variant ^t^ | 2 | (3) | 3 | 3 | N.D. | 0 |
| 5 prime UTR truncation ^u^ | 2 | (2) | 2 | 2 | N.D. | 0 |
| non canonical start codon ^v^ | 1 | (1) | 2 | 2 | N.D. | 2,115 |
| 3 prime UTR truncation ^u^ | 0 | (1) | 1 | 1 | N.D. | 0 |
