# Supplementary material for: Genomic Diversity Evaluation of Populus trichocarpa Germplasm for Rare Variant Genetic Association Studies
Source: Front Genet. 2020 Jan 28;10:1384. doi: 10.3389/fgene.2019.01384 (PMC6997551; doi:10.3389/fgene.2019.01384)
Supplement: Supplementary file 2 [file Table_2.docx]

**Table S2**. Comparison of the number of variants found between variant callers used in this study and two other genomic evaluation studies on *P. trichcocarpa*.

|  | GATK | Platypus | Evans et al. 2014 | BESC |
| --- | --- | --- | --- | --- |
| GATK |  | 8,497,509 | 13,419,720 | 13,339,319 |
| Platypus | 8,497,509 |  | 9,609,166 | 8,892,536 |
| Evans et al. 2014 | 13,419,720 | 9,609,166 |  | N.D |
| BESC | 13,339,319 | 8,892,536 | N.D |  |
